# A Case of Dermatofibrosarcoma Protuberans Arising in a Burn Scar on the Chest Following Electrical Defibrillation

**DOI:** 10.7759/cureus.88438

**Published:** 2025-07-21

**Authors:** Takahiro Hase, Akihiro Orita, Takuya Mizukami, Hiroyuki Nakamura

**Affiliations:** 1 Dermatology, Sapporo City General Hospital, Sapporo, JPN; 2 Dermatology, Kushiro City General Hospital, Kushiro, JPN; 3 Dermatology, JR Sapporo Hospital, Sapporo, JPN

**Keywords:** dermatofibrosarcoma protuberans, electrical burn scar, electrical defibrillation, pedunculated tumor, subcutaneous nodule

## Abstract

Dermatofibrosarcoma protuberans (DFSP) is a rare, locally aggressive skin tumor, and its origin from a burn scar is extremely rare. We report a case of a man in his 60s with DFSP that arose within an electrical burn scar sustained from electrical defibrillation for cardiac arrhythmia 20 years prior. The patient presented with a pedunculated tumor and a nearby subcutaneous nodule on his anterior chest. The diagnosis of DFSP was confirmed by histopathological examination, which revealed a characteristic storiform proliferation of spindle cells, and by immunohistochemistry showing positivity for CD34 and negative for epithelial membrane antigen (EMA), S-100, and alpha-smooth muscle actin (α-SMA). Wide local excision and reconstruction with a latissimus dorsi myocutaneous flap and a split-thickness skin graft were performed. The patient has shown no evidence of recurrence for two years postoperatively. As demonstrated by the subcutaneous nodule in this case, DFSP can be clinically difficult to diagnose. Therefore, careful long-term follow-up and consideration of a skin biopsy, as needed, are crucial for such burn scars.

## Introduction

Dermatofibrosarcoma protuberans (DFSP) is a rare mesenchymal tumor of the skin, with a reported incidence of 0.8 to 4.5 cases per million individuals annually [1]. Characterized by slow but locally aggressive growth and a high propensity for local recurrence, DFSP typically presents as an indurated plaque that may be skin-colored, reddish-brown, or bluish, eventually progressing to a protuberant lesion [1]. It predominantly occurs on the trunk and proximal extremities. Although the precise etiology of DFSP remains incompletely elucidated, prior cutaneous trauma, including surgical scars, vaccination sites, and burn scars, has been implicated as a potential risk factor [2-7]. DFSP arising in the head and neck is a predictor of a poor clinical outcome [1]. To date, four cases of DFSP associated with burn scars have been reported [2-5]. Histopathologically, it is composed of a dense, uniform proliferation of cells with spindle-shaped nuclei, embedded in varying amounts of collagen. This fibroblastic-like proliferation typically forms irregular, interlacing bundles, exhibiting a storiform pattern as seen in many other fibrous proliferations [1]. The mainstay of therapy for localized DFSP is complete surgical resection. Although there is no consensus on the optimal surgical margins, a wide excision of 2-3 cm is associated with a low overall local recurrence rate, ranging from 0% to 30% [1]. We herein report a case of DFSP that developed on the anterior chest in an electrical burn scar sustained during electrical defibrillation approximately 20 years prior.

## Case presentation

**Figure 1:**
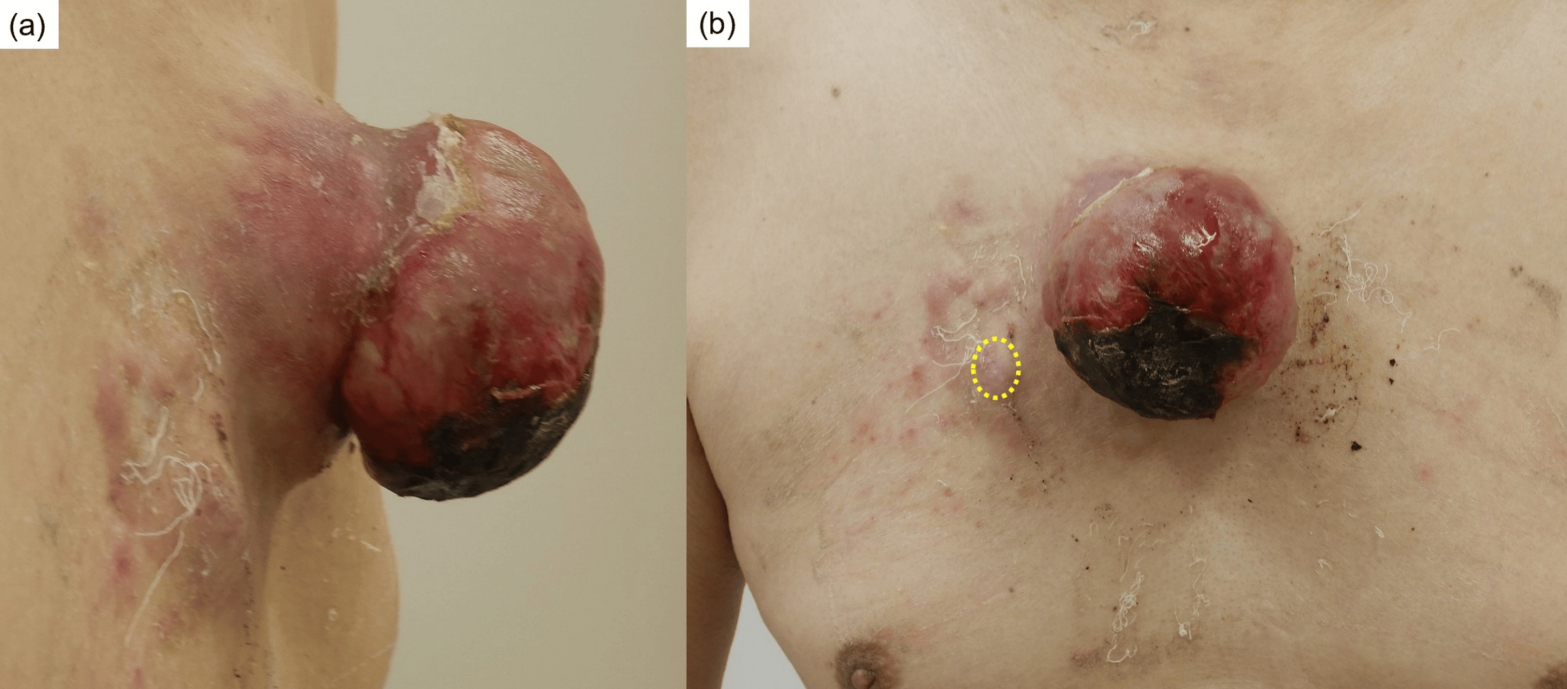
Clinical images. (a) A pedunculated mass, 80 x 80 x 60 mm in size, is present on the anterior chest wall. (b) As indicated by the yellow dotted line, a 20 mm subcutaneous nodule is palpated within the burn scar to the right of the pedunculated mass.

A man in his 60s presented to our department with a tumor on his anterior chest. He reported a history of electrical defibrillation for cardiac arrhythmia in his 40s, which resulted in a burn scar on his chest. Physical examination revealed a pedunculated, elastic, soft tumor measuring 80 × 80 × 60 mm, with an erythematous surface and a partially black eschar. The lesion was non-tender and fixed to the skin. Additionally, a firm, poorly mobile subcutaneous nodule measuring 20 mm in diameter was palpated within the same burn scar (Figure 1).

**Figure 2:**
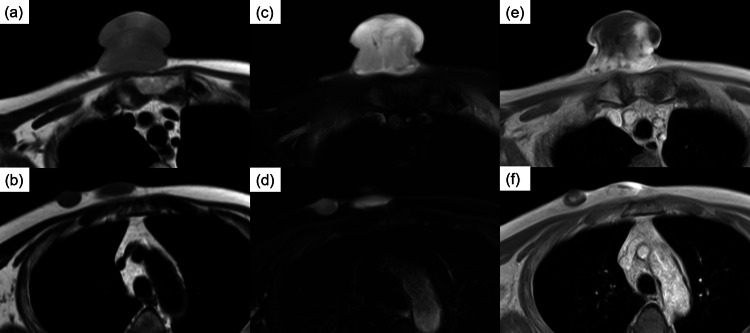
Magnetic resonance imaging (MRI) of the lesions. (a, b) On T1-weighted MRI, the two lesions show low signal intensity. (c, d) On T2-weighted MRI, the two lesions show high signal intensity. (e, f) On contrast-enhanced MRI, heterogeneous enhancement is noted. On imaging, continuity between the two lesions is not clear.

Magnetic resonance imaging (MRI) demonstrated low signal intensity on T1-weighted images and high signal intensity on T2-weighted images for both lesions, with heterogeneous enhancement on contrast-enhanced MRI. No radiological continuity was observed between the two lesions (Figure 2).

**Figure 3:**
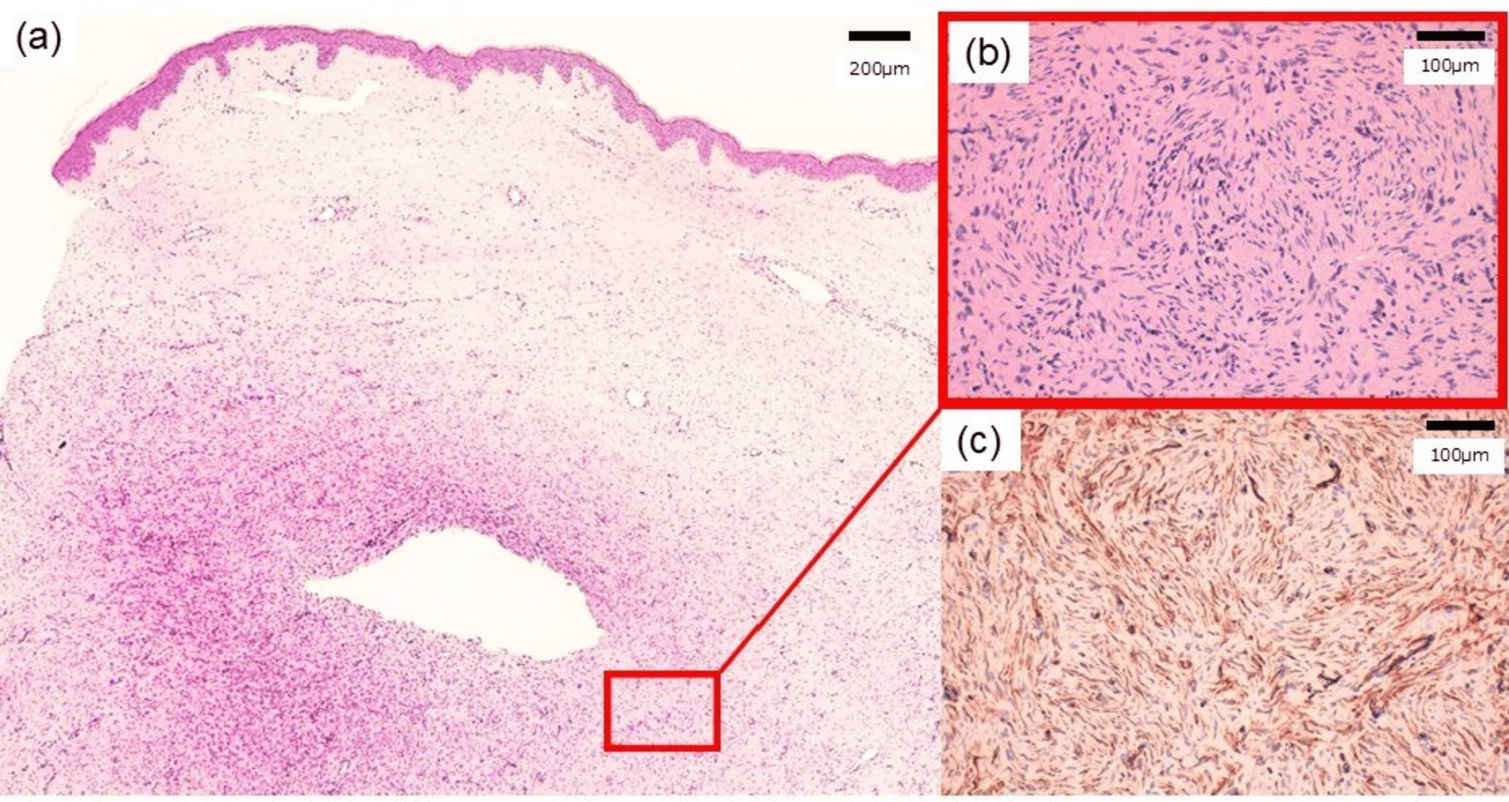
Histopathological findings of the lesion. (a) A proliferation of spindle cells extending from the dermis into the subcutaneous adipose tissue is noted (hematoxylin and eosin staining, x40). (b) A storiform proliferation of spindle cells is noted (hematoxylin and eosin staining, x200). (c) The spindle cells are strongly positive for CD34 (immunohistochemistry, x200).

Skin biopsies were performed on multiple sites, including the main tumor, its neck, and the subcutaneous nodule. Histopathological examination revealed a storiform proliferation of spindle cells extending from the dermis into the subcutaneous adipose tissue. Immunohistochemical analysis confirmed that the spindle cells were positive for CD34 (Figure 3) and negative for epithelial membrane antigen (EMA), S-100, and alpha-smooth muscle actin (α-SMA). Based on these clinical and pathological findings, a diagnosis of DFSP was established.

The patient underwent wide excision with a 20 mm margin and was reconstructed with a latissimus dorsi flap and a split-thickness skin graft. He has remained recurrence-free for two years postoperatively.

## Discussion

DFSP arising in burn scars is extremely rare, and the present case is particularly noteworthy as it developed from an electrical burn scar resulting from a medical procedure, namely, electrical defibrillation. To date, only four cases of DFSP arising in burn scars have been reported (Table 1), none of which occurred in an electrical burn scar. The latency period for the development of post-traumatic DFSP varies, and in cases of DFSP arising from burn scars, it ranges from five to 62 years. While the precise etiology of DFSP remains to be fully elucidated, prior cutaneous trauma has been implicated as a potential risk factor in its development. Several reports and case series have documented the occurrence of DFSP at sites of previous injuries, including surgical scars [6], vaccination sites [7], and burns [2-5]. A characteristic genetic feature of DFSP is a chromosomal translocation involving chromosomes 17 and 22. This translocation t(17;22) results in the fusion of part of the COL1A1 gene on chromosome 17 with part of the PDGFB gene on chromosome 22. Some hypotheses suggest that chronic inflammation and immune system stimulation at the site of trauma may contribute to the malignant transformation of dermal cells [8]. Several other skin malignancies are reported to arise from burn scars, including squamous cell carcinoma, basal cell carcinoma, malignant melanoma, and various sarcomas. Among these, sarcomas are relatively uncommon, reportedly accounting for approximately 5% of such malignancies. DFSP is an even rarer occurrence in this specific context, constituting only about 0.5% of all malignancies developing in burn scars [9]. Although there are few reports of malignancies arising in burn scars after electrical defibrillation, a case of basal cell carcinoma has been reported [10]. In this case, considering the clinical findings and the lesion's origin within a burn scar, the differential diagnosis included squamous cell carcinoma, sarcoma, and pyogenic granuloma. The MRI findings, characterized by low signal intensity on T1-weighted images, high signal intensity on T2-weighted images, and enhancement with a contrast agent, were consistent with DFSP [11]. A subsequent skin biopsy led to the definitive diagnosis of DFSP. While a consensus regarding the optimal surgical margin for DFSP has not been established, a margin of 20 mm or more is frequently recommended in the literature [12]. As the lesion in our case was relatively well-defined, a wide excision was performed with margins exceeding 20 mm. As this case demonstrates, a significant clinical challenge is the difficulty of distinguishing DFSP from benign scar tissue, particularly when it presents as a subcutaneous nodule within a scar. For patients with such burn scars, a skin biopsy should be considered if a suspicious lesion is present.

**Table 1:** Characteristics of dermatofibrosarcoma protuberans arising in burn scars in previous reports and the present case.

Author	Age/sex	Latency period (burn to DFSP diagnosis)	Location	Size	Treatment	Outcome
Bridge et al. [2]	46/M	Approximately 20 years	Left shoulder	80 x 75 x 15 mm	Local excision, including deep fascia and muscle.	Not specified.
Seo et al. [3]	43/M	35 years	Chest	Not specified	Wide excision.	No recurrence at six months post operation.
Tanaka et al. [4]	68/F	62 years	Right axilla	50 x 60 mm	Wide excision.	No recurrence at two years post operation.
Agrawal et al. [5]	32/M	5 years	Right flank	25 x 20 mm	Wide local excision.	Not specified.
Present case	60s/M	Approximately 20 years	Chest	Tumor: 80 x 80 x 60 mm; nodule: 20 mm	Wide local excision with a split-thickness skin graft.	No recurrence at two years postoperatively.

## Conclusions

DFSP can arise from electrical burn scars, including those resulting from medical procedures. A significant clinical challenge is that DFSP, particularly when presenting solely as a subcutaneous nodule within a scar, may be difficult to distinguish from benign scar tissue or other scar-related changes. This underscores the need for careful, long-term follow-up in patients with such scars.
